# Transcriptional Factors and Epigenetic Mechanisms in Obesity and Related Metabolic Comorbidities

**DOI:** 10.3390/cells11162520

**Published:** 2022-08-14

**Authors:** Mohamed Zaiou

**Affiliations:** Institut Jean-Lamour, The University of Lorraine, UMR 7198 CNRS, CEDEX, 54505 Vandoeuvre les Nancy, France; mohamed.zaiou@univ-lorraine.fr

**Keywords:** metabolic diseases, gene expression, type 2 diabetes, DNA methylation, circular RNAs, noncoding RNAs, high fat diet

Recent advances in the study of chromatin remodeling and transcriptional machinery complex dysfunction, and how they drive metabolic-related gene expression have considerably increased our understanding of several molecular processes underlaying obesity and its complications. Obesity is associated with the expansion of adipose tissue, which is induced by an increase in adipogenesis/lipogenesis activity. The adipogenic/lipogenic programs are in part driven by a coordinated activation of transcription factors (TFs) and epigenetic changes. However, the complex working mechanisms of these regulators are far from being clear. 

This editorial summarizes findings from eight original research articles and two review papers assembled in the present Special Issue entitled "Transcriptional Factors and Epigenetic Mechanisms in Obesity and Related Metabolic Comorbidities" (https://www.mdpi.com/journal/cells/special_issues/Transcriptional_Factors, accessed on 10 August 2022). These manuscripts highlight potential roles of specific TFs and/or epigenetic regulatory mechanisms in obesity and related metabolic diseases such as type 2 diabetes mellitus (T2DM) and nonalcoholic fatty liver disease (NAFLD).

Two of the experimental papers addressed the potential metabolic benefits of some medicinal compounds to reduce weight through transcription factors. The first article by Wu and colleagues [1] focuses on how salvia miltiorrhiza extract (SME) and ST32db, a single compound modified from SME, modulate adipogenesis/lipogenesis to prevent high fat diet (HFD)-induced obesity in mice. The study revealed that the later compounds exert their anti-obesity effect through activation of the transcription factor 3 (ATF3)-mediated C/EBPα downregulation and C/EBPα homologous protein (CHOP) pathway. The second article by Colson et al. [2] investigated mechanisms by which arnosic acid (CA), a major compound from rosemary used in traditional medicine, controls brown/brite adipocyte formation and function. Results from this study indicated that CA inhibits browning of white adipocytes and favors decreased gene expression of thermogenic markers by acting as a peroxisome proliferator-activated receptor alpha/gamma (PPARα/γ) antagonist. Therefore, understanding the effect of natural products through TFs regulation may create new avenues for research and opportunities to control fat mass such as in obesity. 

Both overweight and obesity frequently occur with other comorbidities, particularly T2DM. Interferon regulatory factors (IRFs) are emerging as metabolic transcriptional regulators in obesity/T2DM. In this context, Al-Rashed et al. [3] investigated how glucose fluctuations that commonly occur in T2DM patients with poor glycemic control or following intensive therapy, may alter the IRF5 expression and inflammatory responses. Results from this study indicated that the expression of immune-metabolic transcriptional regulator IRF5 is upregulated in monocytes from diabetic patients. Subsequent analysis revealed that the intermittent episodes of hyperglycemia promoted M1 polarization and an inflammatory response via a mechanism involving the toll-like receptor 4 (TLR4)/IRF5 pathway [3]. Obesity is also associated with an increased risk of NAFLD and nonalcoholic steatohepatitis, ultimately leading to hepatocellular carcinoma. In this regard, Tsai et al. [4] attempted to explore the impact of Max dimerization protein 3 (MXD3), a transcription factor that regulates several cellular functions in disorders associated with metabolic diseases. Findings from these studies confirm that Mxd3 promotes obesity through its effect on adipogenesis and could serve as a therapeutic target for obesity-associated liver diseases [4].

TFs are not the only critical players in obesity and associated metabolic disorders. Epigenetic modifiers including DNA methylation, histone modification and noncoding RNAs (ncRNAs) are also implicated in the pathogenesis of obesity and its complications. In addition to altering the compaction of the local chromatin landscape, epigenetic marks can serve as signals to recruit other regulatory factors including transcription factors to influence gene expression. The next papers of this Special Issue highlight examples of the diverse interactions between TFs and epigenetic changes involved in the pathogenesis of obesity. For example, a study by Parrillo and colleagues [5] focused on the role of the transcription factor Homeobox A5 (HOXA5), which is highly expressed in the adipose tissue and plays an active role in regulating adipocyte functions. These authors determined that altered DNA methylation of HOXA5 promoter contributes to restricted adipogenesis in the subcutaneous adipose tissue of lean subjects who were first-degree relatives of T2D subjects and in obese individuals. de la Rocha et al. [6] aimed to study in vivo the effect of arachidonic acid (AA) supplementation for three consecutive generations (prior to coitus in sires or in utero in dams) on phenotypes and epigenetic marks (such as gene specific DNA methylation) of offspring mice. Results from this work indicated that body weight and global DNA methylation cumulatively increase in F2 and F3 offspring generations and positively correlate with paternal or maternal offspring AA exposure. Authors speculated that parental mice nutrition affects offspring metabolism through epigenetic modifications of specific genes across generation. Findings from a study by Hajri et al. [7] demonstrated that both HFD and palmitic acid alter global and *pparγ*-promoter DNA methylation, leading to enhanced fat accumulation in the liver, an initiation step in the development of NAFLD. These results provide strong evidence that modification of the *pparγ* promoter methylation is a crucial mechanism of regulation in NAFLD pathogenesis. Previous studies have suggested that obesity and HFD induced insulin resistance contribute to the dysfunction of salivary glands. Thus, saliva may be promising avenue to monitor health status, disease onset, and progression. Four and a half LIM domains 2 (FHL2) is a key regulator of intracellular signal transduction pathways and the *fhl2* gene is consistently found as one of the top hyper-methylated genes upon aging. Remarkably, FHL2 expression increases with methylation. This was demonstrated in relevant metabolic tissues including white adipose tissue, pancreatic β-cells, and skeletal muscle. Here, a review article by Habibe et al. [8] provided an overview of the current knowledge on regulation of FHL2 by genetic variation and epigenetic DNA modification and the relevance of this molecule in aging and age-related diseases, including obesity and T2DM. Additional example of the interaction between TFs and epigenetics came from a study by Huang et al. [9] showing that microRNA (miR)-22-3p/Sp1 pathway plays an important role in modulating claudin-1 and claudin-3 expression in the diabetic submandibular glands of a T2D mouse model. Further results from this study indicated that the transcription factor Sp1 was a specific target of miR-22-3p and that high glucose reduced the interaction between miR-22-3p and Sp1. Finally, circular RNAs, as class of ncRNAs, emerge as promising prognostic biomarkers for diagnosis of obesity and related metabolic disorders as reviewed comprehensively by Zaiou [10]. The author explored the current stage of knowledge on these RNA species and their implication in adipogenesis and obesity, providing an updated analysis of their potential to serve as diagnostic and therapeutic tools. 

To sum up, the above promising findings suggest that TFs and epigenetic changes are key players in metabolic-related gene regulation, therefore, can serve as an attractive target for effective treatment for metabolic diseases including obesity. However, further research into the complex interactions between the genome, environment, and epigenetic marks, is required in this field.
